# Effects of mowing on body size patterns in spider assemblages of mesic meadows

**DOI:** 10.1002/ece3.10892

**Published:** 2024-02-15

**Authors:** Tomasz Stański, Marzena Stańska, Izabela Hajdamowicz, Łukasz Nicewicz, Andreas Hiller

**Affiliations:** ^1^ Faculty of Sciences University of Siedlce Siedlce Poland; ^2^ Flächenagentur Baden‐Württemberg GmbH Ostfildern Germany; ^3^ Faculty of Natural Sciences, Institute of Biology, Biotechnology and Environmental Protection University of Silesia Katowice Poland; ^4^ Independent Researcher Wendlingen Germany

**Keywords:** agriculture, agroecosystems, Araneae, epigeic spiders, habitat structure, semi‐natural meadows

## Abstract

Habitat disturbance affects not only the abundance, species richness and species composition of the local fauna, but also the body size of specific individuals and body size patterns in animal assemblages. Particularly large disturbances occur in agroecosystems, where many agricultural treatments are carried out. One of them, which is most commonly applied to grasslands and which significantly damages the habitat structure, is mowing. We examined the effect of mowing on mean, skewness and kurtosis of the body size in epigeic spider assemblages. The research was conducted on mesic meadows in eastern Poland, in an agricultural landscape typical for this region, consisting of a mosaic of meadows, fields and forests. Spiders were collected using pitfall traps in two sampling periods: the first before mowing and the second when part of the meadows had been mown. Mowing had no significant effect on mean body size, skewness and kurtosis of the body size in epigeic spider assemblages. However, after the cut, mown plots showed, on average, significantly smaller spider species than unmown plots. Both the value of skewness and kurtosis significantly increased after mowing but to the same extent on both the control and mown plots. The decrease in mean body size and increase in skewness in spider assemblages were mainly due to an increase in the number of small species from the Linyphiidae family. It is likely that these species began to migrate (via ballooning) during the second sampling session, following the start of haying, and were thus caught in traps more frequently. Our study showed no clear, significant changes in the body size structure of epigeic spiders in mown meadows compared to unmown ones, which may suggest that the mowing, where extensive farming is practised, does not have a long‐term significant negative impact on this group of invertebrates.

## Graphical abstract

In the submitted paper, we described how mowing, as a strong habitat disturbance, influenced on mean, skewness and kurtosis of the body size in epigeic spider assemblages. We found that mowing had no significant effect on mean body size, skewness and kurtosis of the body size in epigeic spider assemblages. However, after the cut, mown plots showed, on average, significantly smaller spider species than unmown plots. Both the value of skewness and kurtosis significantly increased after mowing but to the same extent on both the control and mown plots.

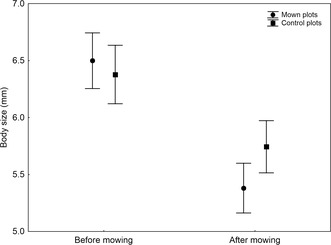

## 1 INTRODUCTION

Body size is an important trait that largely determines many ecological characteristics of animals, such as their distribution, ability to disperse and reproduce, energy use, extinction risk, as well as interactions with other animals or trophic position (Arim et al., 2010; Ernest, 2005; Fattorini et al., 2013; Fenchel & Finlay, 2004; Peters, 1983; Ripple et al., 2017). Both many ecosystem processes and habitat conditions affect not only the body size of specific individuals but are also reflected in species body size distribution in animal assemblages. Therefore, certain regularities regarding body size patterns can be observed within them. For example, the frequency of small versus large species may increase towards moist and more complex habitats due to, respectively, their lower resistance to desiccation and more fractal structure of the environment, which provides more space available for small animals (DeVito & Formanowicz, 2003; Entling et al., 2010; Gunnarsson, 1992; Morse et al., 1985). Furthermore, there are geographical patterns indicating an increase in the average body size of species recorded in assemblages with increasing latitude, as was shown in the case of ants (Cushman et al., 1993) or, conversely, a decrease as was shown for spiders (Entling et al., 2010).

Human activity, causing among other things climate change and considerable transformation of many habitats, is currently one of the most significant factors affecting animal assemblages (e.g. Crooks et al., 2017; Graham et al., 2019; Thomas et al., 2004). A whole range of human‐induced changes affect not only the abundance, species richness or species composition of local fauna but also the body size of specific individuals of animals and body size patterns in their assemblages (Hamřik et al., 2023; Lomolino & Perault, 2007; Senior et al., 2013). For example, climate change causes a reduction in body size in assemblages as a result of shrinking body sizes of individual species or the loss of large species (Gardner et al., 2011; Sheridan & Bickford, 2011). Similarly, forest fragmentation can lead to a decrease in body size, as demonstrated by Lomolino and Perault (2007) for small mammals. In addition, urbanisation can increase the proportion of small species, as was shown for beetle assemblages (Magura et al., 2004). Also, the introduction of alien species into habitats can disrupt specific body size patterns, for example, modify latitudinal patterns of Bergmann's rule, as was the case in fish assemblages (Blanchet et al., 2010). Therefore, the study of body size patterns may contribute to the understanding of how animal assemblages respond to changes in their habitat, which can be important for biodiversity conservation. Despite this, research on how human‐induced disturbance alters body size patterns is rare and the mechanisms are still poorly understood.

Human disturbance is particularly severe in agroecosystems where many agricultural treatments are implemented. One of the most destructive treatments to habitat structure, and one that is most commonly used and has a strong impact on resident organisms, is mowing. This treatment can cause significant declines in the abundance, density and diversity of many groups, such as orthopterans, spiders, butterflies and beetles (Humbert et al., 2010; Mazalová et al., 2015; Nyffeler & Breene, 1990; Rada et al., 2014; Thorbek & Bilde, 2004). However, detailed knowledge of how mowing affects body size patterns in animal assemblages on meadows is lacking, as studies on this subject are scarce (Birkhofer et al., 2015; Hamřik & Košulic, 2021).

Spiders, as animals whose escape ability is much lower compared to flying arthropods, are highly vulnerable to the negative effects of mowing (Mazalová et al., 2015). Their abundance can decrease by up to 50% within a week after grass cutting, both as a direct result of killing during mowing and as a result of migration to new habitats (Thorbek & Bilde, 2004). Spiders are abundant invertebrate predators in agroecosystems and play a key role in pest control, thus detailed knowledge of how mowing affects their assemblages, including body size patterns, can be very helpful for sustainable and efficient agriculture (Nyffeler & Sunderland, 2003).

The main objective of our study was to test whether mowing, as a habitat disturbance that significantly alters habitat structure, affects body size patterns in spider assemblages. To investigate body size patterns, we used three metrics calculated based on the body size of species recorded in a given assemblage: (1) mean body size, (2) skewness and (3) kurtosis.

We hypothesised that mowing, by simplifying the habitat structure, favours larger species, thus the mean body size of spider assemblages would be higher after this treatment, while the skewness would decrease due to the lower proportion of small species. Our second hypothesis was that mowing would increase the value of kurtosis, that is, a given spider assemblage would include species whose body size would deviate less from the mean body size calculated for the whole assemblage of the mown meadows compared to that of the unmown meadows. Our assumptions were based on the results of Gibb et al. (2018), who showed for ants that both the largest and smallest species (i.e. specialists) disappear in disturbed habitats, because they are replaced by more medium‐sized, and thus generalist species. In addition, we thought that in more complex habitats (unmown meadows), there are potentially more diverse niches that support spider assemblages with greater species size diversity compared to simplified habitats (mown meadows).

## 2 MATERIALS AND METHODS

### 2.1 Study area

**FIGURE 1:**
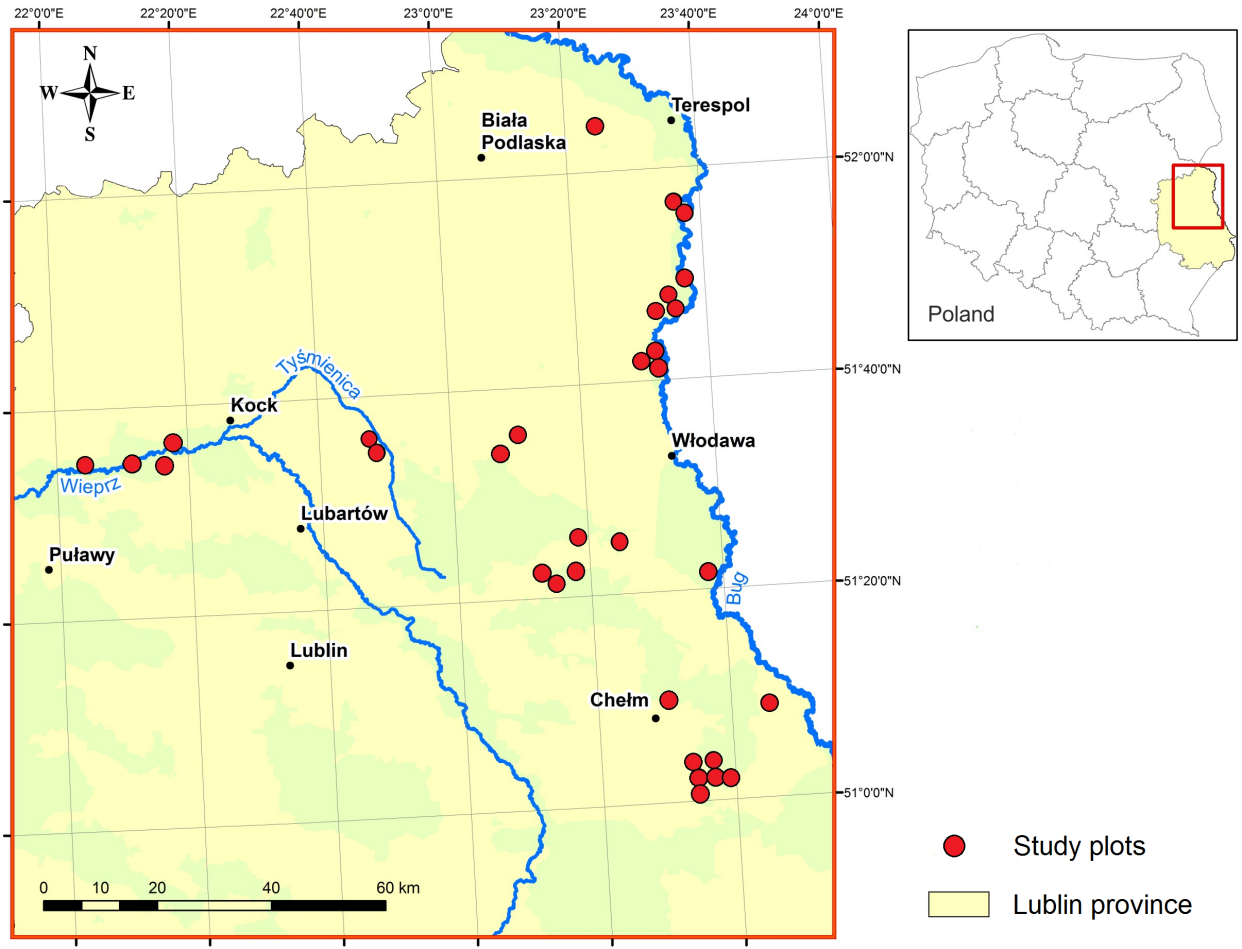
Location of the study plots.

The research was conducted from 2013 to 2015 in eastern Poland, in the Lublin Province, which is dominated by an agricultural landscape consisting of a mosaic of meadows, fields and forests. We selected 32 plots located in semi‐natural mesic meadows (Figure 1) that are highly productive and at the same time valuable grasslands in terms of nature. Vegetation height and density in all study plots were similar and the plant species in the meadows were mostly the same. The dominant plant species growing on these plots were sod grasses, mainly tall oat‐grass (*Arrhenatherum elatius*) and orchard grass (*Dactylis glomerata*), forming the highest layer of sward (with an average height of 45–50 cm in the first sampling session, and 60–70 cm in the second sampling session on unmown plots). Grasses were accompanied by many species of dicotyledon plants such as common hogweed (*Heracleum sphondylium*), wild carrot (*Daucus carota*), field scabious (*Knautia arvensis*) and goat's beard (*Tragopogon orientalis*). The lowest layer of vegetation consisted of species such as common bird's‐foot trefoil (*Lotus corniculatus*), lesser trefoil (*Trifolium dubium*), red clover (*T. pratense*), white clover (*T. repens*), common yarrow (*Achillea millefolium*), oxeye daisy (*Leucanthemum vulgare*) and spreading bellflower (*Campanula patula*). Extensive farming was performed on the plots, and the meadows were mown only once or twice a year, with the first cut starting in the third decade of May. All cuts were made using a rotary mower and the vegetation reached a height of 5–15 cm after mowing.

### 2.2 Spider sampling

Spiders were collected on each study plot each year in two 14‐day sampling periods. The first sampling period was carried out before the mowing and lasted from the second decade of May to early June, while the second sampling period was conducted when part of the meadows had been mown and lasted from mid‐June to the end of the first decade of July. Spiders were sampled using pitfall traps, which were plastic cups with a diameter of 8.4 cm shielded by a roof to protect them from rain and prevent small mammals from falling inside. Each trap was filled to one‐third of its volume with propylene glycol as a killing and preservative agent. Three pitfall traps operated on each plot, placed in a straight line every 10 m. They were usually located in the central part of the meadow, at least 20 m from the edge, to avoid collecting spiders in the ecotone zone. Spiders collected on a given meadow in a given year (in the first and second period, forming pairs of data) were treated as a separate sample. Some samples had to be excluded from the analysis due to trap damage caused by water flooding or by animals (mainly wild boar). A total of 80 samples were collected, with 42 samples from plots that were mown after the first sampling period and 38 samples from plots that remained unmown. Unmown plots were treated as the control plots, while those that were mown after the first sampling period were treated as the mown plots.

### 2.3 Data analysis

The collected material was sorted in the laboratory and the spiders contained were identified to species level. To investigate the effect of mowing on body size patterns, we calculated the mean, skewness and kurtosis of the body size for spider assemblages on each plot in each sampling period separately, based on the list of recorded species. As a body size, we used the maximum body length value for each species regardless of sex, provided in the Spiders of Europe database (Nentwig et al., 2023). Positive (right) skewness reflects a higher frequency of small species in a given assemblage relative to large ones, whereas negative (left) skewness indicates the predominance of large species. Low kurtosis indicates a larger number of outliers in a dataset, while high kurtosis indicates a smaller number of such observations, and thus that the results are more clustered around the mean. To test whether mowing affected the body size, skewness and kurtosis determined for spider assemblages on mesic meadows, two‐way repeated ANOVA was used. The mean body size, skewness and kurtosis calculated for species found in the first and second sampling period on a given plot were treated as repeated measures. Species richness can significantly affect the body size distribution in spider assemblages and thus may be a major factor explaining specific body size patterns within them (Meiri & Thomas, 2007), so we tested whether the number of recorded spider species was correlated with mean body size, skewness and kurtosis using Kendall's rank correlation. Statistical analysis was performed using software STATISTICA 12.0.

## 3 RESULTS

A total of 25,317 spider individuals belonged to 139 species and 18 families were collected during the study. During the first sampling period (before mowing) a total of 8375 individuals were collected on the control plots and 9914 individuals on the mown plots. During the second sampling period (after mowing), the number of collected spiders was 3707 and 3321 respectively. The mean number of spider species collected before mowing was 17.7 ± 4.6 SD (min. 9, max 28) on the Control plots and 16.5 ± 4.5 SD (min 7, max 28) on the mown plots while after mowing it was 16.4 ± 5.0 SD (min 4, max 29) and 14.6 ± 3.6 SD (min 8, max 22) respectively. The largest number of species found on both types of study plots before mowing belonged to the family Lycosidae, while after mowing, the number of species from the family Linyphiidae was equally high. Altogether, spiders from these two families accounted for an average of 60–70% of all species found on each type of plot and in each sampling period (Table 1).

**TABLE 1:** The mean number and mean percent of species from individual families collected on control and mown plots before mowing and after mowing.

Family	Before mowing				After mowing			
	Control plots		Mown plots		Control plots		Mown plots	
	*N*	%	*N*	%	*N*	%	*N*	%
Gnaphosidae	1.1	6.4	1.0	6.1	1.3	7.8	0.5	3.6
Linyphiidae	3.4	18.1	3.5	20.1	5.2	31.2	5.7	39.1
Lycosidae	7.0	40.8	7.1	44.9	6.1	38.2	5.1	34.7
Tetragnathidae	1.4	7.9	1.5	8.8	0.9	5.8	1.3	9.1
Thomisidae	2.1	11.7	1.6	9.7	1.2	8.3	1.2	7.9
Other	2.7	15.1	1.8	10.4	1.6	8.7	0.8	5.5
Total	17.7	100	16.5	100	16.4	100	14.6	100

*Note*: Only the five most numerous families in terms of number of species captured were listed. ‘Other’ include families: Araneidae, Clubionidae, Dictynidae, Eutichuridae, Hahniidae, Mimetidae, Miturgidae, Philodromidae, Phrurolithidae, Pisauridae, Salticidae, Theridiidae.

**FIGURE 2:**
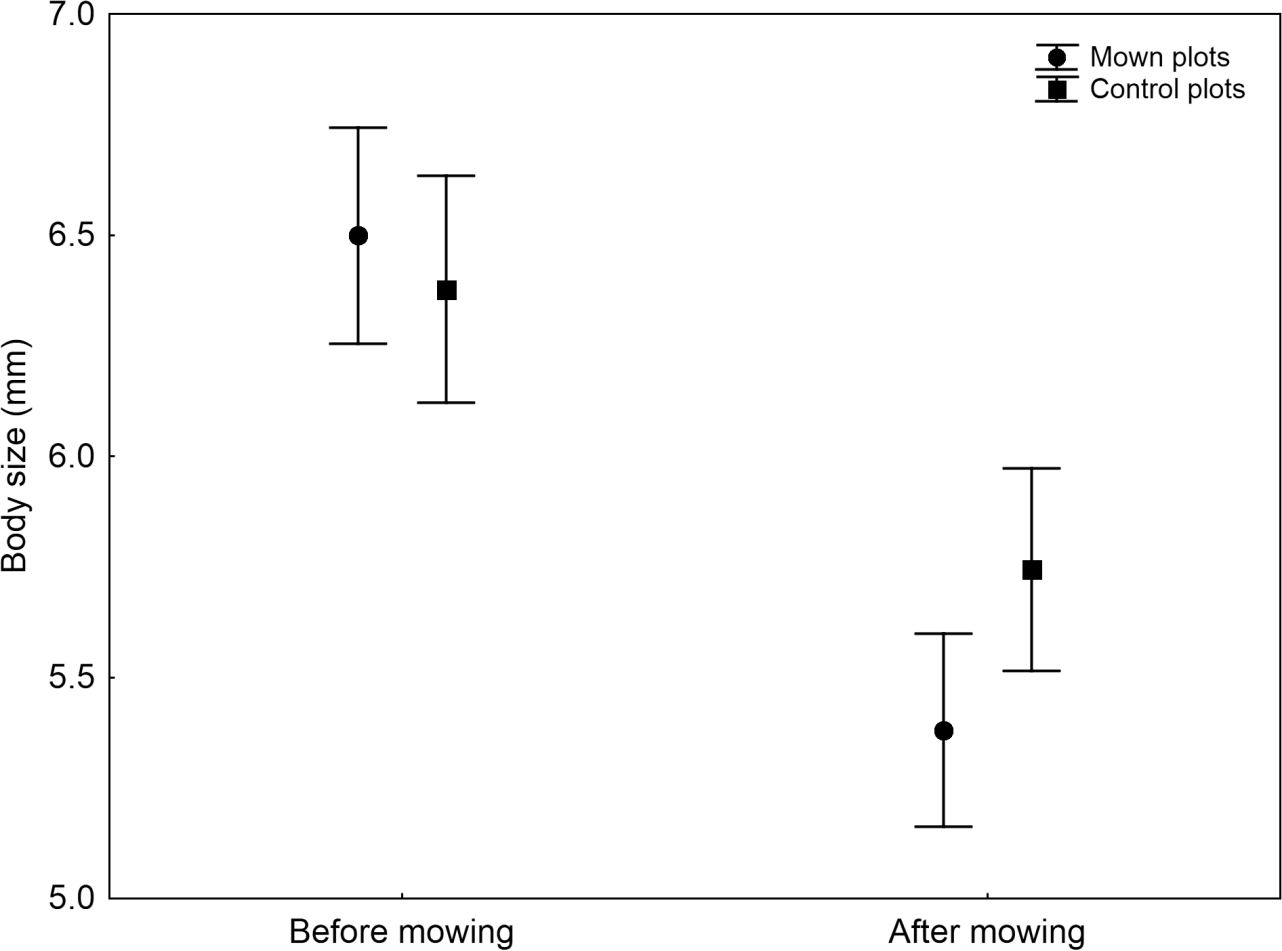
The mean body size (with 95% confidence limits) in spider assemblages on control and mown plots before mowing and after mowing.

**FIGURE 3:**
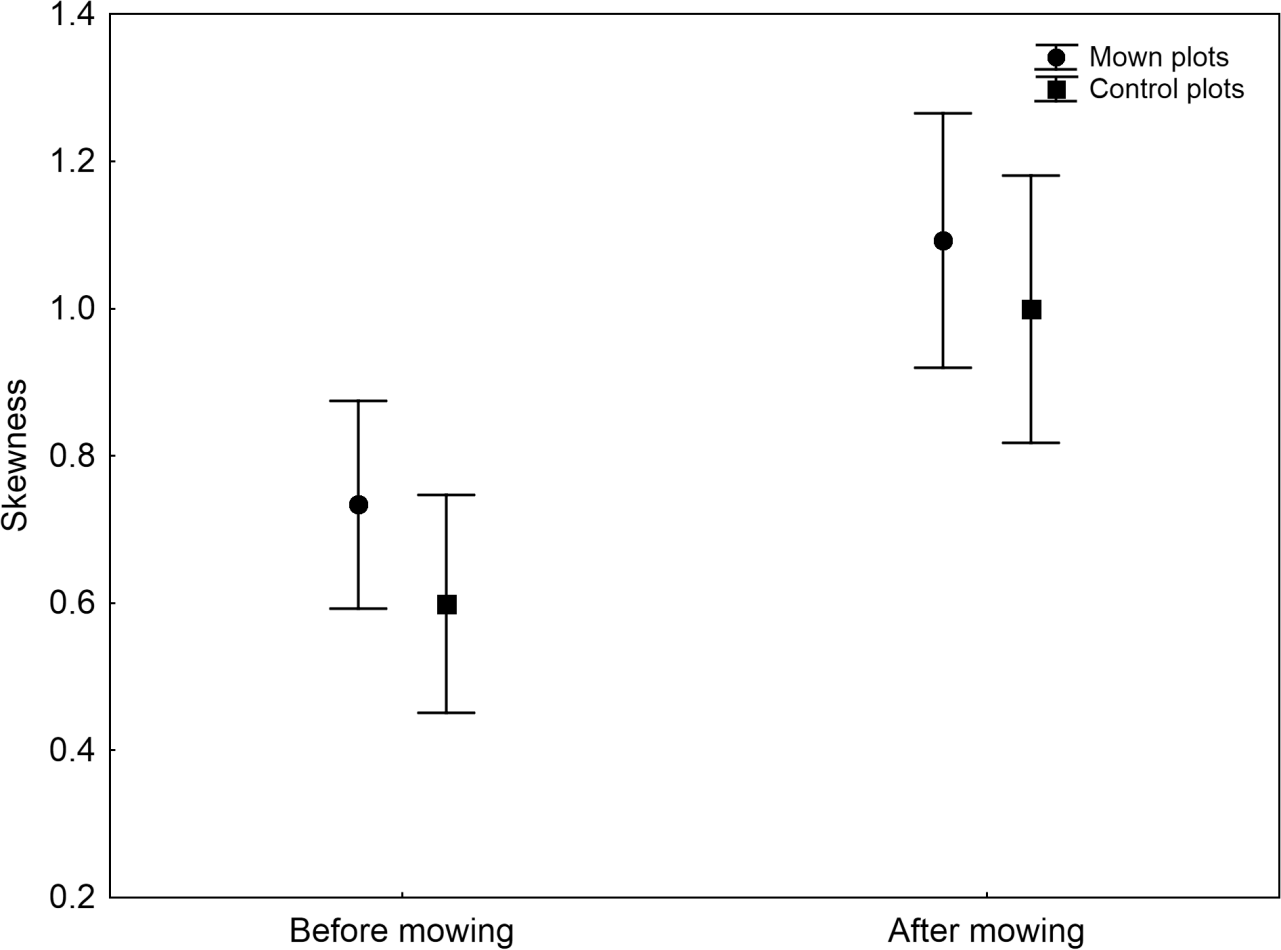
The mean skewness (with 95% confidence limits) of body size in spider assemblages on control and mown plots before mowing and after mowing.

**FIGURE 4:**
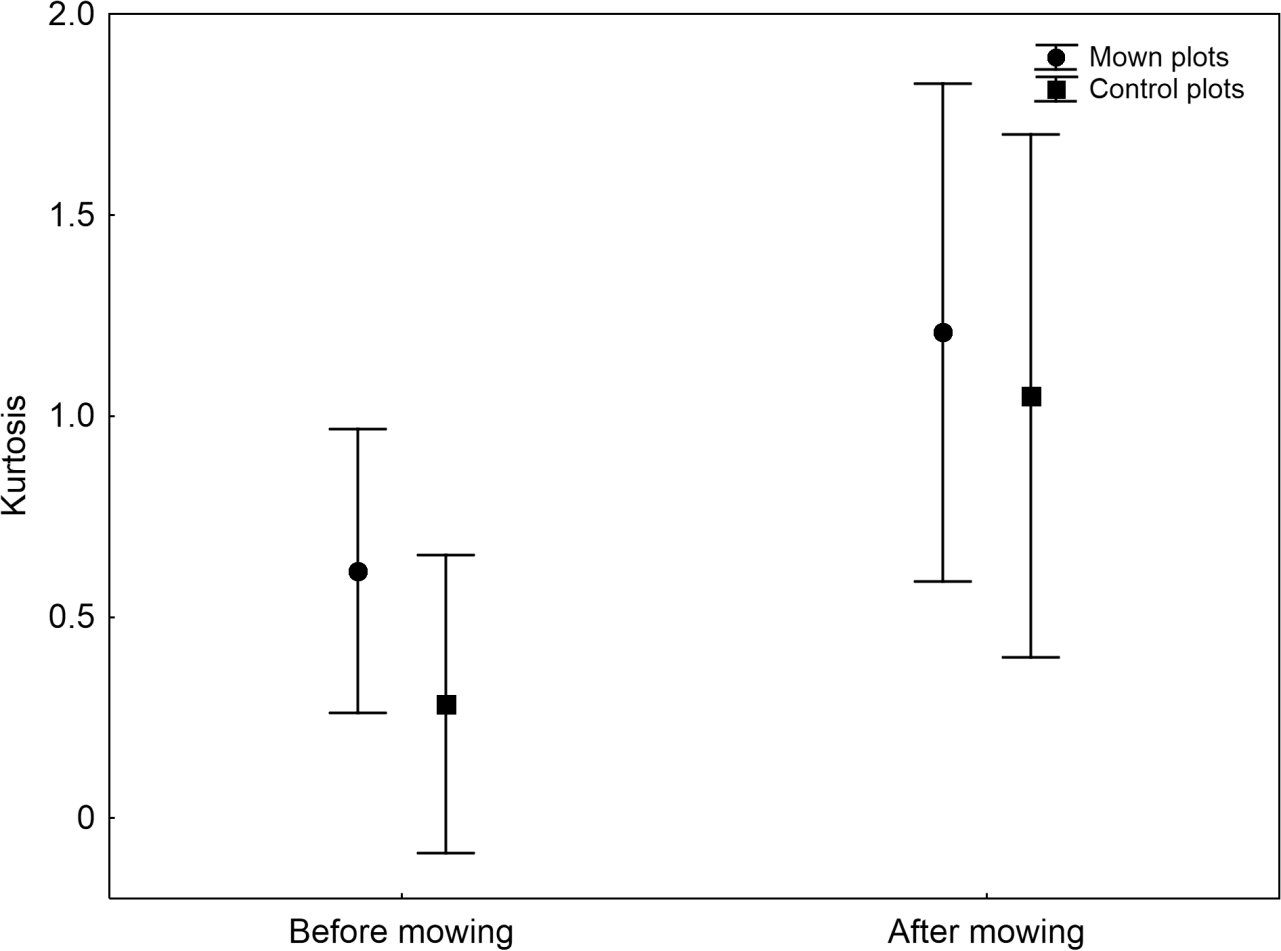
The mean kurtosis (with 95% confidence limits) of body size in spider assemblages on control and mown plots before mowing and after mowing.

Mowing had no significant direct effect on mean body size, skewness and kurtosis (Table 2). However, after the cut, mown plots showed on average significantly smaller spider species than unmown plots (Figure 2). Both the skewness and kurtosis significantly increased after mowing but to the same extent on both control and mown plots (Figures 3 and 4). These patterns were independent of the number of species because the number of species did not correlate significantly with body size (Kendall's rank correlation, *Z* = 0.03, *p* = .978, *τ* = 0.001), skewness (*Z* = 0.29, *p* = .774, *τ* = 0.015) and kurtosis (*Z* = 0.40, *p* = .690, *τ* = 0.021).

**TABLE 2:** Results of the analysis of variance (ANOVA) testing the effects of treatment (mown plots vs. control plots), sampling period (before mowing vs. after mowing), and their interaction on the mean body size, skewness and kurtosis of spider assemblages on mesic meadows.

Effect	df	Body size			Skewness			Kurtosis		
		MS	*F*	*p*	MS	*F*	*p*	MS	*F*	*p*
Intercept	1	5750.23	9114.63	**<.000**	113.70	359.22	**<.000**	89.12	30.45	**<.000**
Treatment	1	0.64	1.02	.315	0.64	2.03	.158	3.23	1.10	.300
Error (Pair)	78	0.63			0.32			2.93		
Sampling period	1	30.50	62.58	**<.000**	6.12	30.64	**<.000**	21.06	8.84	**.004**
Sampling period × treatment	1	2.49	5.18	**.026**	0.02	0.10	.755	0.33	0.14	.709
Error (Residuals)	78	0.49			0.20			2.38		

*Note*: Significant results are shown in bold.

## 4 DISCUSSION

Our study showed no clear, significant differences in body size patterns of epigeic spiders between mown meadows and unmown ones. We found that the effect of mowing on body size patterns manifested itself only in the fact that the decrease in body size in the second sampling period was greater on the mown plots than on the control plots. Our hypothesis that mowing favours larger species, causing an increase in mean body size and a decrease in skewness (i.e. decrease in the proportion of small species) in spider assemblages, was not confirmed. When formulating this hypothesis, we relied on several phenomena. First of all, mowing drastically simplifies the structure of habitat by significantly reducing the height of plants, which translates into changes in microclimatic conditions such as temperature, which increases, and humidity, which decreases (Gardiner & Hassall, 2009; Guido & Gianelle, 2001). As large spiders have greater resistance to desiccation compared to small spiders, which lose water at a higher rate, thus reducing their survival in dry environments (DeVito & Formanowicz, 2003; Vincent, 1993), wet (unmown) habitats should be more abundant in smaller spider species compared to dry (mown) habitats. In addition, habitats with more complex structure support higher species richness, species diversity and a larger number of individuals than habitats with simpler structure, and thus the former provide more food (Diehl et al., 2013; Gardner et al., 1995; Hauser et al., 2006). Given the phenomenon that small organisms, in general, are less resistant to lack of food, disturbances that simplify habitat structure primarily expose small species to starvation, thus favouring larger species (Cushman et al., 1993; Gergs & Jager, 2014). Moreover, the results of studies by other authors showed that more complex habitats support smaller species, due to the larger space available for small organisms compared to less complex habitats (Gunnarsson, 1992; Hutchinson & MacArthur, 1959; Morse et al., 1985). We also considered the fact that the main predators of spiders are other spiders, and cannibalism and intraguild predation significantly affect spider assemblages (Wagner & Wise, 1996; Wise, 2006). Intraguild predation and cannibalism are stronger in simple habitats, while more complex habitats reduce these phenomena, which promotes the coexistence of these predators (Finke & Denno, 2006; Langellotto & Denno, 2006). Since smaller spiders in general are more likely to fall prey to larger ones (Rypstra & Samu, 2005), the chance for survival of small spiders in more complex habitats is higher.

Instead of the expected increase in body size and decrease in skewness of body size in spider assemblages on the mown plots in the second sampling session, which could be indicated by the phenomena described above, we obtained opposite results for both analysed groups of meadows. We found that the proportion of small species increased in the second sampling period, which was mainly due to the presence of the Linyphiidae family, represented by many small species, which became more abundant compared to the first sampling period. On the other hand, the number and proportion of species from the Lycosidae family, which comprises species of large size, decreased in the second sampling period, especially on the mown plots. It is the larger proportion of species from the family Linyphiidae and the smaller proportion from the family Lycosidae in the second sampling period in the mown meadows compared to the control meadows that were mainly responsible for the significant difference in body size found between these two groups of meadows.

The larger number of species from the family Linyphiidae caught in pitfall traps during the second sampling period compared to the first one is probably due to the start of haying in the study area. Pitfall trapping is a popular method for studying epigeic invertebrate assemblages, but the number of specimens collected does not only reflect the abundance of a given species but also its activity (Topping & Sunderland, 1992). It is possible that spiders of the family Linyphiidae became more active during the second sampling period and were thus caught in traps more frequently. In addition, the commencement of swathing not only in the study meadows but also in other meadows, even those located much farther away, may have caused spiders of this family to leave the threatened sites and spread by ballooning. It has been shown that ballooning is particularly important for the survival of spiders (including the Linyphiidae family) in disturbed and unpredictable habitats, such as agroecosystems (Halley et al., 1996; Thomas & Jepson, 1999; Weyman et al., 2002).

Another likely reason for the greater decrease in body size in spider assemblages we found on the mown plots compared to the control plots could be predation by birds. Meadows after cutting are attractive for many bird species as foraging sites (Peggie et al., 2011). These predators are often selective in choosing their prey, and the selection may include such features as colour, individual behaviour, sex or size (Gaston et al., 1997; Gunnarsson, 2007). As predators that use their eyesight to search for prey, birds usually pick larger (i.e. more conspicuous) spiders (Askenmo et al., 1977; Gunnarsson, 1996, 1998). Moreover, large prey has a higher energy value compared to small prey, thus birds should prefer large spiders also for energy reasons. Therefore, a larger size increases the risk of becoming prey for predators and consequently, large species may be eliminated in habitats with higher predation pressure, such as meadows after grass cutting.

Our second hypothesis was that kurtosis increased on the mown plots in the second sampling period due to the elimination of the largest and smallest spider species from the assemblages. Gibb et al. (2018) suggested that species at the extremes of the size scale as specialist predators in disturbed habitats are replaced by medium‐sized species that are generalist predators. This is because diet specialisation limits the ability of species to respond to changes in food availability, thus increasing the risk of their extinction (Brook et al., 2008; Davies et al., 2004). Although spiders are generalist predators, feeding on a wide range of prey, their trophic niche and feeding behaviour are determined by, among others, their body size (Sanders et al., 2015). Moreover, the body size of spiders is closely related to the body size of prey they capture, which may determine the type of food they feed on (Nentwig & Wissel, 1986; Nyffeler, 1999). For example, detrital prey like collembolans is essential for relatively small spiders (Shimazaki & Miyashita, 2005). In our study, however, we observed an increase in values of kurtosis for both mown and control plots. This may suggest that spider assemblages respond not only to changes occurring directly in their habitat but also to those occurring in larger area. It is possible that the start of haymaking in neighbouring meadows affects also meadows that have not yet been cut, as both spiders and their potential prey migrate from the former to the latter, altering spider assemblages in terms of their composition and abundance (Thorbek & Bilde, 2004). Our study was conducted in a fragmented agricultural landscape, which is a mosaic of meadows, fields and forests, and where haying in adjacent meadows starts at different times. It is, therefore, very likely that grass cutting in one meadow can significantly affect invertebrate assemblages inhabiting adjacent meadows.

The main limitation of our study was that the body sizes for the spider species were based on literature (Nentwig et al., 2023). A study based on measured, actual body sizes of spiders captured in the study plots would perhaps better show the effect of mowing on body size patterns in spider assemblages. However, such a methodology to study body size patterns, based on body size data obtained from the literature or online databases, has been widely used by many other authors both for invertebrates (e.g. Ulrich, 2006; Ulrich & Szpila, 2008) and vertebrates (e.g. Coetzee et al., 2013; Rodríguez et al., 2006). In the case of spiders, Entling et al. (2010) used such a methodology to investigate the relationship between spider body size and climate variables in Europe. Furthermore, our work was limited to only one method of capturing spiders, that is, pitfall traps. The inclusion of other methods, for example, sweep net, would have given a more complete picture of the impact of mowing on body size patterns in different groups of spiders inhabiting meadows. Mowing may have different effects on different groups of spiders, varying not so much in their taxonomic affiliation as in characteristics such as body size or hunting behaviour. A group that is undoubtedly significantly affected by mowing are web‐building spiders. Mowing, by destroying structures for web attachment, forces them to migrate in search of new habitats (i.e. unmown meadows) where structures sufficient to build webs still exist. In contrast, the effect of mowing on the abundance of wandering spiders seems to be less pronounced. Hamřik and Košulic (2021), studying different conservation management methods in xeric grasslands, found that mown and burnt patches hosted species with smaller mean body size than unmanaged patches and patches, where turf was mechanically disturbed. However, this phenomenon was only observed in the case of vegetation‐dwelling spiders; in the case of ground‐dwelling spiders, this effect was not observed.

In many studies, the negative effect of mowing on spider assemblages was demonstrated (Baines et al., 1998; Mazalová et al., 2015; Thorbek & Bilde, 2004). Based on our results, we cannot conclude that mowing has a negative effect on epigeic spider assemblages. However, the greater decrease in body size found in mown plots compared to control plots may suggest that such an effect could potentially exist. Perhaps it is only visible for a short period of time, immediately after mowing. The capture of the spiders in our study lasted for 2 weeks and it seems that during this time the spiders were able to recolonise the mown grasslands. This was helped by the fragmented landscape, and that both the study meadows and the meadows adjacent to the study plots were mowed at different times due to the fact that they belonged to multiple landowners who did not coordinate agricultural activities. This allows spiders to escape to unmown areas where spiders can hide after mowing and from where they migrate to recolonise the mown areas. The above‐mentioned suggestions can be used as recommendations for grassland management. On the other hand, for studies in an area with large‐scale mowing, one would expect different results due to the limited number of refugia for spiders and fewer opportunities for recolonisation of mowed areas.

In conclusion, we found that mowing had no significant direct effect on mean body size, skewness and kurtosis. The direct effect of mowing on body size patterns is manifested only in the fact that, in the second sampling period, the decrease in body size in spider assemblages on mown plots was greater than the decrease on the control plots. At this time, the number of small species from the family Linyphiidae increased on both meadows, but to a greater extent on the mown plots. Both the value of skewness and the value of kurtosis of body size in spider assemblages significantly increased in the second sampling period compared to the first one, but to the same extent on both control and mown plots. Our results may suggest that spider assemblages respond not only to changes occurring directly in their habitat but also to those occurring in the surrounding environment, that is, in neighbouring meadows where mowing has partially started.

## AUTHOR CONTRIBUTIONS

**Tomasz Stański:** Conceptualization (equal); formal analysis (lead); methodology (equal); visualization (lead); writing – original draft (equal); writing – review and editing (equal). **Marzena Stańska:** Conceptualization (equal); funding acquisition (lead); methodology (equal); project administration (lead); writing – original draft (equal); writing – review and editing (equal). **Izabela Hajdamowicz:** Conceptualization (equal); funding acquisition (supporting); methodology (equal); writing – review and editing (equal). **Łukasz Nicewicz:** Investigation (equal); writing – review and editing (equal). **Andreas Hiller:** Investigation (equal); writing – review and editing (equal).

## CONFLICT OF INTEREST STATEMENT

The authors declare no conflict of interest.

## Data Availability

All data are archived in the Dryad Digital Repository (https://doi.org/10.5061/dryad.b8gtht7k).

## References

[ece310892-bib-0001] Arim, M. , Abades, S. R. , Laufer, G. , Loureiro, M. , & Marquet, P. A. (2010). Food web structure and body size: Trophic position and resource acquisition. Oikos, 119, 147–153. 10.1111/j.1600-0706.2009.17768.x

[ece310892-bib-0066] Askenmo, C. , von Brömssen, A. , Ekman, J. , & Jansson, C. (1977). Impact of some wintering birds on spider abundance in spruce. Oikos, 28, 90–94. 10.2307/3543327

[ece310892-bib-0002] Baines, M. , Hambler, C. , Johnson, P. J. , Macdonald, D. W. , & Smith, H. (1998). The effects of arable field margin management on the abundance and species richness of Araneae (spiders). Ecography, 21, 74–86.

[ece310892-bib-0003] Birkhofer, K. , Diekötter, T. , Meub, C. , Stötzel, K. , & Wolters, V. (2015). Optimizing arthropod predator conservation in permanent grasslands by considering diversity components beyond species richness. Agriculture, Ecosystems and Environment, 211, 65–72. 10.1016/j.agee.2015.05.014

[ece310892-bib-0004] Blanchet, S. , Grenouillet, G. , Beauchard, O. , Tedesco, P. A. , Leprieur, F. , Dürr, H. H. , Busson, F. , Oberdorff, T. , & Brosse, S. (2010). Non‐native species disrupt the worldwide patterns of freshwater fish body size: Implications for Bergmann's rule. Ecology Letters, 13, 421–431. 10.1111/j.1461-0248.2009.01432.x 20100241

[ece310892-bib-0005] Brook, B. W. , Sodhi, N. S. , & Bradshaw, C. J. A. (2008). Synergies among extinction drivers under global change. Trends in Ecology & Evolution, 23, 453–460. 10.1016/j.tree.2008.03.011 18582986

[ece310892-bib-0006] Coetzee, B. W. T. , le Roux, P. C. , & Chown, S. L. (2013). Scale effects on the body size frequency distributions of African birds: Patterns and potential mechanisms. Global Ecology and Biogeography, 22, 380–390. 10.1111/j.1466-8238.2012.00793.x

[ece310892-bib-0007] Crooks, K. R. , Burdett, C. L. , Theobald, D. M. , King, S. R. , Di Marco, M. , Rondinini, C. , & Boitani, L. (2017). Quantification of habitat fragmentation reveals extinction risk in terrestrial mammals. Proceedings of the National Academy of Sciences of the United States of America, 114, 7635–7640. 10.1073/pnas.1705769114 28673992 PMC5530695

[ece310892-bib-0008] Cushman, J. H. , Lawton, J. H. , & Manly, B. F. J. (1993). Latitudinal patterns in European ant assemblages: Variation in species richness and body size. Oecologia, 95, 30–37. 10.1007/BF0064950 28313308

[ece310892-bib-0009] Davies, K. F. , Margules, C. R. , & Lawrence, J. F. (2004). A synergistic effect puts rare, specialized species at greater risk of extinction. Ecology, 85, 265–271. 10.1890/03-0110

[ece310892-bib-0010] DeVito, J. , & Formanowicz, D. R. (2003). The effects of size, sex, and reproductive condition on thermal and desiccation stress in a riparian spider (*Pirata sedentarius*, Araneae, Lycosidae). The Journal of Arachnology, 31, 278–284. 10.1636/02-20

[ece310892-bib-0011] Diehl, E. , Mader, V. L. , Wolters, V. , & Birkhofer, K. (2013). Management intensity and vegetation complexity affect web‐building spiders and their prey. Oecologia, 173, 579–589. 10.1007/s00442-013-2634-7 23494286

[ece310892-bib-0012] Entling, W. , Schmidt‐Entling, M. H. , Bacher, S. , Brandl, R. , & Nentwig, W. (2010). Body size‐climate relationships of European spiders. Journal of Biogeography, 37, 477–485. 10.2307/25654266

[ece310892-bib-0013] Ernest, S. K. M. (2005). Body size, energy use, and community structure of small mammals. Ecology, 86, 1407–1413. 10.1890/03-3179

[ece310892-bib-0014] Fattorini, S. , Sciotti, A. , Tratzi, P. , & Di Giulio, A. (2013). Species distribution, ecology, abundance, body size and phylogeny originate interrelated rarity patterns at regional scale. Journal of Zoological Systematics and Evolutionary Research, 51, 279–286. 10.1111/jzs.12026

[ece310892-bib-0015] Fenchel, T. , & Finlay, B. J. (2004). The ubiquity of small species: Patterns of local and global diversity. Bioscience, 54, 777–784. 10.1641/0006-3568(2004)054[0777:TUOSSP]2.0.CO;2

[ece310892-bib-0061] Finke, D. L. , & Denno, R. F. (2006). Spatial refuge from intraguild predation: implications for prey suppression and trophic cascades. Oecologia, 149, 265–275. 10.1007/s00442-006-0443-y 16708227

[ece310892-bib-0016] Gardiner, T. , & Hassall, M. (2009). Does microclimate affect grasshopper populations after cutting of hay in improved grassland? Journal of Insect Conservation, 13, 97–102. 10.1007/s10841-007-9129-y

[ece310892-bib-0017] Gardner, J. L. , Peters, A. , Kearney, M. R. , Joseph, L. , & Heinsohn, R. (2011). Declining body size: A third universal response to warming? Trends in Ecology & Evolution, 26, 285–291. 10.1016/j.tree.2011.03.005 21470708

[ece310892-bib-0018] Gardner, S. M. , Cabido, M. R. , Valladares, G. R. , & Diaz, S. (1995). The influence of habitat structure on arthropod diversity in Argentine semi‐arid Chaco forest. Journal of Vegetation Science, 6, 349–356. 10.2307/3236234

[ece310892-bib-0064] Gaston, K. J. , Chown, S. L. , & Styles, C. V. (1997). Changing size and changing enemies: the case of the mopane worm. Acta Oecologica, 18, 21–26. 10.1016/S1146-609X(97)80077-X

[ece310892-bib-0019] Gergs, A. , & Jager, T. (2014). Body size‐mediated starvation resistance in an insect predator. Journal of Animal Ecology, 83, 758–768. 10.1111/1365-2656.12195 24417336

[ece310892-bib-0021] Gibb, H. , Sanders, N. J. , Dunn, R. R. , Arnan, X. , Vasconcelos, H. L. , Donoso, D. A. , Andersen, A. N. , Silva, R. R. , Bishop, T. R. , Gomez, C. , Grossman, B. F. , Yusah, K. M. , Luke, S. H. , Pacheco, R. , Pearce‐Duvet, J. , Retana, J. , Tista, M. , & Parr, C. L. (2018). Habitat disturbance selects against both small and large species across varying climates. Ecography, 41, 1184–1193. 10.1111/ecog.03244

[ece310892-bib-0022] Graham, S. I. , Kinnaird, M. F. , O'Brien, T. G. , Vågen, T.‐G. , Winowiecki, L. A. , Young, T. P. , & Young, H. S. (2019). Effects of land‐use change on community diversity and composition are highly variable among functional groups. Ecological Applications, 29, 1973. 10.1002/eap.1973 31306541

[ece310892-bib-0023] Guido, M. , & Gianelle, D. (2001). Distribution patterns of four orthoptera species in relations to microhabitat heterogeneity in an ecotonal area. Acta Oecologica, 22, 175–185. 10.1016/S1146-609X(01)01109-2

[ece310892-bib-0024] Gunnarsson, B. (1992). Fractal dimension of plants and body size distribution in spiders. Functional Ecology, 6, 636–641. 10.2307/2389957

[ece310892-bib-0067] Gunnarsson, B. (1996). Bird predation and vegetation structure affecting spruce‐living arthropods in a temperate forest. Journal of Animal Ecology, 65, 389–397. 10.2307/5885

[ece310892-bib-0068] Gunnarsson, B. (1998). Bird predation as a sex‐ and size‐selective agent of the arboreal spider *Pityohyphantes phrygianus* . Functional Ecology, 12, 453–458.

[ece310892-bib-0065] Gunnarsson, B. (2007). Bird predation on spiders: ecological mechanisms and evolutionary consequences. Journal of Arachnology, 35, 509–529. 10.1636/RT07-64.1

[ece310892-bib-0025] Halley, J. M. , Thomas, C. F. G. , & Jepson, P. C. (1996). A model for the spatial dynamics of linyphiid spiders in farmland. Journal of Applied Ecology, 33, 471–492. 10.2307/2404978

[ece310892-bib-0026] Hamřik, T. , & Košulic, O. (2021). Impact of small‐scale conservation management methods on spider assemblages in xeric grassland. Agriculture, Ecosystems & Environment, 307, 107225. 10.1016/j.agee.2020.107225

[ece310892-bib-0027] Hamřik, T. , Košulic, O. , Gallé, R. , Gallé‐Szpisjak, N. , & Hédl, R. (2023). Opening the canopy to restore spider biodiversity in protected oakwoods. Forest Ecology and Management, 541, 121064. 10.1016/j.foreco.2023.121064

[ece310892-bib-0028] Hauser, A. , Attrill, M. J. , & Cotton, P. A. (2006). Effects of habitat complexity on the diversity and abundance of macrofauna colonising artificial kelp holdfasts. Marine Ecology Progress Series, 325, 93–100. 10.3354/meps325093

[ece310892-bib-0029] Humbert, J.‐Y. , Ghazoul, J. , Richner, N. , & Walter, T. (2010). Hay harvesting causes high orthopteran mortality. Agriculture, Ecosystems and Environment, 139, 522–527. 10.1016/j.agee.2010.09.012

[ece310892-bib-0030] Hutchinson, G. E. , & MacArthur, R. H. (1959). A theoretical ecological model of size distributions among species of animals. The American Naturalist, 93, 117–125.

[ece310892-bib-0062] Langellotto, G. A. , & Denno, R. F. (2006). Refuge from cannibalism in complex‐structured habitats: implications for the accumulation of invertebrate predators. Ecological Entomology, 31, 575–581. 10.1111/j.1365-2311.2006.00816.x

[ece310892-bib-0031] Lomolino, M. V. , & Perault, D. R. (2007). Body size variation of mammals in a fragmented, temperate rainforest. Conservation Biology, 21, 1059–1069. 10.1111/j.1523-1739.2007.00727.x 17650255

[ece310892-bib-0032] Magura, T. , Tóthmérész, B. , & Molnár, T. (2004). Changes in carabid beetle assemblages along an urbanisation gradient in the city of Debrecen, Hungary. Landscape Ecology, 19, 747–759. 10.1007/s10980-005-1128-4

[ece310892-bib-0033] Mazalová, M. , Šipoš, J. , Rada, S. , Kašák, J. , Šarapatka, B. , & Kuras, T. (2015). Responses of grassland arthropods to various biodiversity‐friendly management practices: Is there a compromise? European Journal of Entomology, 112, 734–746. 10.14411/eje.2015.076

[ece310892-bib-0059] Meiri, S. , & Thomas, G. H. (2007). The geography of body size – challenges of the interspecific approach. Global Ecology and Biogeography, 16, 689–693. 10.1111/j.1466-8238.2007.00343.x

[ece310892-bib-0034] Morse, D. R. , Lawton, J. H. , Dodson, M. M. , & Williamson, M. H. (1985). Fractal dimension of vegetation and the distribution of arthropod body lengths. Nature, 314, 731–733. 10.1038/314731a0

[ece310892-bib-0035] Nentwig, W. , Blick, T. , Bosmans, R. , Gloor, D. , Hänggi, A. , & Kropf, C. (2023). Spiders of Europe. https://www.araneae.nmbe.ch/

[ece310892-bib-0036] Nentwig, W. , & Wissel, C. (1986). A comparison of prey lengths among spiders. Oecologia, 68, 595–600. 10.1007/BF00378777 28311718

[ece310892-bib-0037] Nyffeler, M. (1999). Prey selection of spiders in the field. Journal of Arachnology, 27, 317–324.

[ece310892-bib-0038] Nyffeler, M. , & Breene, R. G. (1990). Spiders associated with selected European hay meadows, and the effects of habitat disturbance, with the predation ecology of the crab spiders, *Xysticus* spp. (Araneae, Thomisidae). Journal of Applied Entomology, 110, 149–159. 10.1111/j.1439-0418.1990.tb00108.x

[ece310892-bib-0039] Nyffeler, M. , & Sunderland, K. D. (2003). Composition, abundance and pest control potential of spider communities in agroecosystems: A comparison of European and US studies. Agriculture, Ecosystems and Environment, 95, 579–612. 10.1016/S0167-8809(02)00181-0

[ece310892-bib-0040] Peggie, C. T. , Garratt, C. M. , & Whittingham, M. J. (2011). Creating ephemeral resources: How long do the beneficial effects of grass cutting last for birds? Bird Study, 58, 390–398. 10.1080/00063657.2011.597841

[ece310892-bib-0041] Peters, R. H. (1983). The ecological implications of body size. Cambridge University Press.

[ece310892-bib-0043] Rada, S. , Mazalová, M. , Šipoš, J. , & Kuras, T. (2014). Impacts of mowing, grazing and edge effect on orthoptera of submontane grasslands: Perspectives for biodiversity protection. Polish Journal of Ecology, 62, 123–138. 10.3161/104.062.0112

[ece310892-bib-0044] Ripple, W. J. , Wolf, C. , Newsome, T. M. , Hoffmann, M. , Wirsing, A. J. , & McCauley, D. J. (2017). Extinction risk is most acute for the world's largest and smallest vertebrates. Proceedings of the National Academy of Sciences, 114, 10678–10683. 10.1073/pnas.1702078114 PMC5635868 28923917

[ece310892-bib-0045] Rodríguez, M. Á. , López‐Sañudo, I. L. , & Hawkins, B. A. (2006). The geographic distribution of mammal body size in Europe. Global Ecology and Biogeography, 15, 173–181. 10.1111/j.1466-822X.2006.00206.x

[ece310892-bib-0063] Rypstra, A. L. , & Samu, F. (2005). Size dependent intraguild predation and cannibalism in coexisting wolf spiders (Araneae, Lycosidae). Journal of Arachnology, 33, 390–397. 10.1636/CT05-10.1

[ece310892-bib-0046] Sanders, D. , Vogel, E. , & Knop, E. (2015). Individual and species‐specific traits explain niche size and functional role in spiders as generalist predators. Journal of Animal Ecology, 84, 134–142. 10.1111/1365-2656.12271 25041766

[ece310892-bib-0047] Senior, M. J. M. , Hamer, K. C. , Bottrell, S. , Edwards, D. P. , Fayle, T. M. , Lucey, J. M. , Mayhew, P. J. , Newton, R. , Peh, K. S.‐H. , Sheldon, F. H. , Stewart, C. , Styring, A. R. , Thom, M. D. F. , Woodcock, P. , & Hill, J. K. (2013). Trait‐dependent declines of species following conversion of rain forest to oil palm plantations. Biodiversity and Conservation, 22, 253–268. 10.1007/s10531-012-0419-7

[ece310892-bib-0048] Sheridan, J. A. , & Bickford, D. (2011). Shrinking body size as an ecological response to climate change. Nature Climate Change, 1, 401–406. 10.1038/nclimate1259

[ece310892-bib-0049] Shimazaki, A. , & Miyashita, T. (2005). Variable dependence on detrital and grazing food webs by generalist predators: Aerial insects and web spiders. Ecography, 28, 48–494. 10.1111/j.0906-7590.2005.04105.x

[ece310892-bib-0050] Thomas, C. D. , Cameron, A. , Green, R. E. , Bakkenes, M. , Beaumont, L. J. , Collingham, Y. C. , Erasmus, B. F. N. , Ferreira de Siqueira, M. , Grainger, A. , Hannah, L. , Hughes, L. , Huntley, B. , van Jaarsveld, A. S. , Midgley, G. F. , Miles, L. , Ortega‐Huerta, M. A. , Townsend Peterson, A. , Phillips, O. L. , & Williams, S. E. (2004). Extinction risk from climate change. Nature, 427, 145–148. 10.1038/nature02121 14712274

[ece310892-bib-0051] Thomas, C. F. G. , & Jepson, P. C. (1999). Differential aerial dispersal of linyphiid spiders from a grass and a cereal field. Journal of Arachnology, 27, 294–300.

[ece310892-bib-0052] Thorbek, P. , & Bilde, T. (2004). Reduced numbers of generalist arthropod predators after crop management. Journal of Applied Ecology, 41, 526–538. 10.1111/j.0021-8901.2004.00913.x

[ece310892-bib-0053] Topping, C. J. , & Sunderland, K. D. (1992). Limitations to the use of pitfall traps in ecological studies exemplified by a study of spiders in a field of winter wheat. Journal of Applied Ecology, 29, 485–491. 10.2307/2404516

[ece310892-bib-0054] Ulrich, W. (2006). Body size distribution of European Hymenoptera. Oikos, 114, 518–528. 10.1111/j.2006.0030-1299.14839.x

[ece310892-bib-0055] Ulrich, W. , & Szpila, K. (2008). Body size distributions of eastern European Diptera. Polish Journal of Ecology, 56, 557–568.

[ece310892-bib-0056] Vincent, L. S. (1993). The natural history of the California turret spider *Atypoides riversi* (Araneae, Antrodiaetidae): Demographics, growth rates, survivorship, and longevity. Journal of Arachnology, 21, 29–39.

[ece310892-bib-0057] Wagner, J. D. , & Wise, D. H. (1996). Cannibalism regulates densities of young wolf spiders: Evidence from field and laboratory experiments. Ecology, 77, 639–652. 10.2307/2265637

[ece310892-bib-0058] Weyman, G. S. , Sunderland, K. D. , & Jepson, P. C. (2002). A review of the evolution and mechanisms of ballooning by spiders inhabiting arable farmland. Ethology Ecology & Evolution, 14, 307–326. 10.1080/08927014.2002.9522733

[ece310892-bib-0060] Wise, D. H. (2006). Cannibalism, food limitation, intraspecific competition, and the regulation of spider populations. Annual Review of Entomology, 51, 441–465. 10.1146/annurev.ento.51.110104.150947 16332219

